# Exploring the mechanism of transformation in *Acacia nilotica* (Linn.) triggered by colchicine seed treatment

**DOI:** 10.1186/s12870-024-05139-9

**Published:** 2024-05-21

**Authors:** Muhammad Wasim Haider, Muhammad Nafees, Rashid Iqbal, Habat Ullah Asad, Farrukh Azeem, Muhammad Samsam Raza, Abdel-Rhman Z. Gaafar, Mohamed S. Elshikh, Muhammad Arslan, Muhammad Habib Ur Rahman, Ayman M. S. Elshamly

**Affiliations:** 1 Department of Horticultural Sciences, Faculty of Agriculture and Environment, The Islamia University of Bahawalpur, Bahawalpur, 63100 Pakistan; 2 Department of Agronomy, Faculty of Agriculture and Environment, The Islamia University of Bahawalpur Pakistan, Bahawalpur, 63100 Pakistan; 3 Centre for Agriculture and Bioscience International, Rawalpindi, 46300 Pakistan; 4 Agri Development, Fauji Fresh N Freeze Ltd, Gulberg II, Lahore, 48000 Pakistan; 5 Department of Botany and Microbiology, College of Science, King Saud University, Riyadh, 11451 Saudi Arabia; 6 Institute of Crop Science and Resource Conservation (INRES), Crop Science, University of Bonn, 53115 Bonn, Germany; 7 Department of Agroecology and Organic Farming, Institute of Crop Science and Resource Conservation (INRES), University of Bonn, Bonn, 53115 Germany; 8 Water Studies and Research Complex, National Water Research Center, Cairo, Egypt

**Keywords:** *Acacia nilotica*Linn., Colchicine, Enzymatic and non-enzymatic antioxidants, Germination percentage, Morphology, Stomatal index, Reactive oxygen species, Photosynthesis-related metrics, Seed treatment

## Abstract

**Background:**

*Acacia nilotica* Linn. is a widely distributed tree known for its applications in post-harvest and medicinal horticulture. However, its seed-based growth is relatively slow. Seed is a vital component for the propagation of *A. nilotica* due to its cost-effectiveness, genetic diversity, and ease of handling. Colchicine, commonly used for polyploidy induction in plants, may act as a pollutant at elevated levels. Its optimal concentration for *Acacia nilotica*'s improved growth and development has not yet been determined, and the precise mechanism underlying this phenomenon has not been established. Therefore, this study investigated the impact of optimized colchicine (0.07%) seed treatment on *A. nilotica*'s morphological, anatomical, physiological, fluorescent, and biochemical attributes under controlled conditions, comparing it with a control.

**Results:**

Colchicine seed treatment significantly improved various plant attributes compared to control. This included increased shoot length (84.6%), root length (53.5%), shoot fresh weight (59.1%), root fresh weight (42.8%), shoot dry weight (51.5%), root dry weight (40%), fresh biomass (23.6%), stomatal size (35.9%), stomatal density (41.7%), stomatal index (51.2%), leaf thickness (11 times), leaf angle (2.4 times), photosynthetic rate (40%), water use efficiency (2.2 times), substomatal CO_2_ (36.6%), quantum yield of photosystem II (13.1%), proton flux (3.1 times), proton conductivity (2.3 times), linear electron flow (46.7%), enzymatic activities of catalase (25%), superoxide dismutase (33%), peroxidase (13.5%), and ascorbate peroxidase (28%), 2,2-diphenyl-1-picrylhydrazyl-radical scavenging activities(23%), total antioxidant capacity (59%), total phenolic (23%), and flavonoid content (37%) with less number of days to 80% germination (57.1%), transpiration rate (53.9%), stomatal conductance (67.1%), non-photochemical quenching (82.8%), non-regulatory energy dissipation (24.3%), and H_2_O_2_ (25%) and O^−2^ levels (30%).

**Conclusion:**

These findings elucidate the intricate mechanism behind the morphological, anatomical, physiological, fluorescent, and biochemical transformative effects of colchicine seed treatment on *Acacia nilotica* Linn. and offer valuable insights for quick production of *A. nilotica*’s plants with modification and enhancement from seeds through an eco-friendly approach.

**Supplementary Information:**

The online version contains supplementary material available at 10.1186/s12870-024-05139-9.

## Background

The Acacia genus, belongs to the Leguminosae family (Mimosideae subfamily and Acacieae tribe) and encompasses approximately 1200 to 1300 species which are predominantly found in the subtropical regions [1]. *Acacia nilotica* Linn. exhibits a prolific distribution across the continents of Africa, Americas, Asia, and Australia [2]. The ploidy levels in this group of species vary from 2n = 2x = 26 to 2n = 8x = 104. *A. nilotica*, with a ploidy level of 2n = 2x = 26, exhibits slow growth when raised from the seed but this species occupies a significant place for its biomass applications in horticulture [3]. For instance, Arabic gum, which is extracted from the stems of Acacia spp. trees, has been widely documented to reduce the respiration rate and ethylene production in fruits and vegetables which enhances the products' resistance to phytopathogens and delays the fruit senescence [4–7]. Furthermore, numerous bioactive compounds have also been extracted from various parts of *A. nilotica*, encompassing apigenin, catechin, gallic acid, kaempferol, niloticane, rutin, umbelliferone, as well as two steroids, namely β-sitosterol and rostene; which indicate the significance of its utilization in the medicine. Nonetheless, different species of Acacia have been tested for their increased production potential [8]. But this process requires a significant time and resources to conduct comprehensively.

In recent studies, colchicine has been found to have a substantial influence on morphological, biochemical and anatomical traits of different plants species which ultimately determine the effectiveness of their fluorescence and physiological activities [9]. However, it is highly toxic to human health and unsafe to the environment at elevated levels [10]. In plants, the elevated levels of colchicine also lead to deformities and diminish their growth and productivity [11]. So, the determination of optimum colchicine concentration is of dire need for seed treatment of plants to alter the morphological traits *i.e.,* plant size, leaf thickness, and leaf colour [12, 13]. The use of colchicine below toxic level for extended periods may be a reliable strategy to mitigate its harmful effects and boost the crop growth and production rate. Numerous previous studies report the application of 0.1% colchicine in orchid for 96 h [14], 1% colchicine in lily for 24 h [15], 0.05% colchicine in salvia for 24 h [16], 0.2% colchicine in Japanese barberry for 24 h [17], 0.5% colchicine in phlox for 36 h [18], 0.08% colchicine in calendula for 4 h [19], 0.2% colchicine in marigold for 3 h [20], 0.2% colchicine in rose for 12 h [21], and 0.1–0.3% colchicine in gladiolus for 24 h [22] for their successful growth and development.

Morphological and physiological changes in plants may occur by application of colchicine due to an increase in cell size, gene silencing and epigenetic or genetic interactions [23–25]. Additionally, an improvement in the resistibility of plants to the environmental stresses and diseases have also been observed [26]. Another significant advantage associated with the application of colchicine is that it facilitates the acquisition of novel functions by the mutant without compromising its essential functions [27]. The impact of colchicine on polyploidy induction and its mechanism has been widely studied but there is no literature found that signifies its influence on growth and development of tree species particularly *A. nilotica*. Therefore, this study was carried out to investigate the changes in plant morphological, anatomical, physiological, fluorescent, and biochemical attributes of *A. nilotica* Linn.

## Results

### Morphometric and anatomical attributes of *Acacia nilotica* Linn

**Fig. 1:**
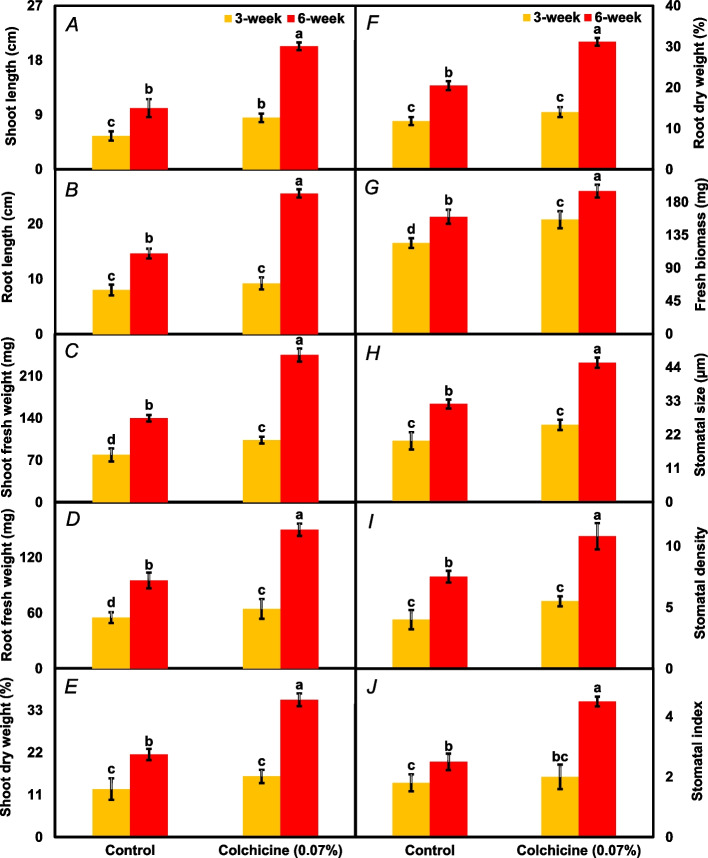
The morphological and anatomical attributes including shoot length (**A**), root length (**B**), shoot fresh weight (**C**), shoot dry weight (**D**), root fresh weight (**E**), root dry weight (**F**), fresh biomass (**G**), stomatal size (**H**), stomatal density (**I**), and stomatal index (**J**) recorded in *Acacia nilotica*’ s seedlings raised under colchicine seed treatment and control. The bars indicate the standard error ( ±) of the mean (*n* = 3). Lettering denotes statistical variations between the treatment means carried out using Least Significant Difference Test at the *P* ≤ *0.05* after analysis of variance

**Fig. 2:**
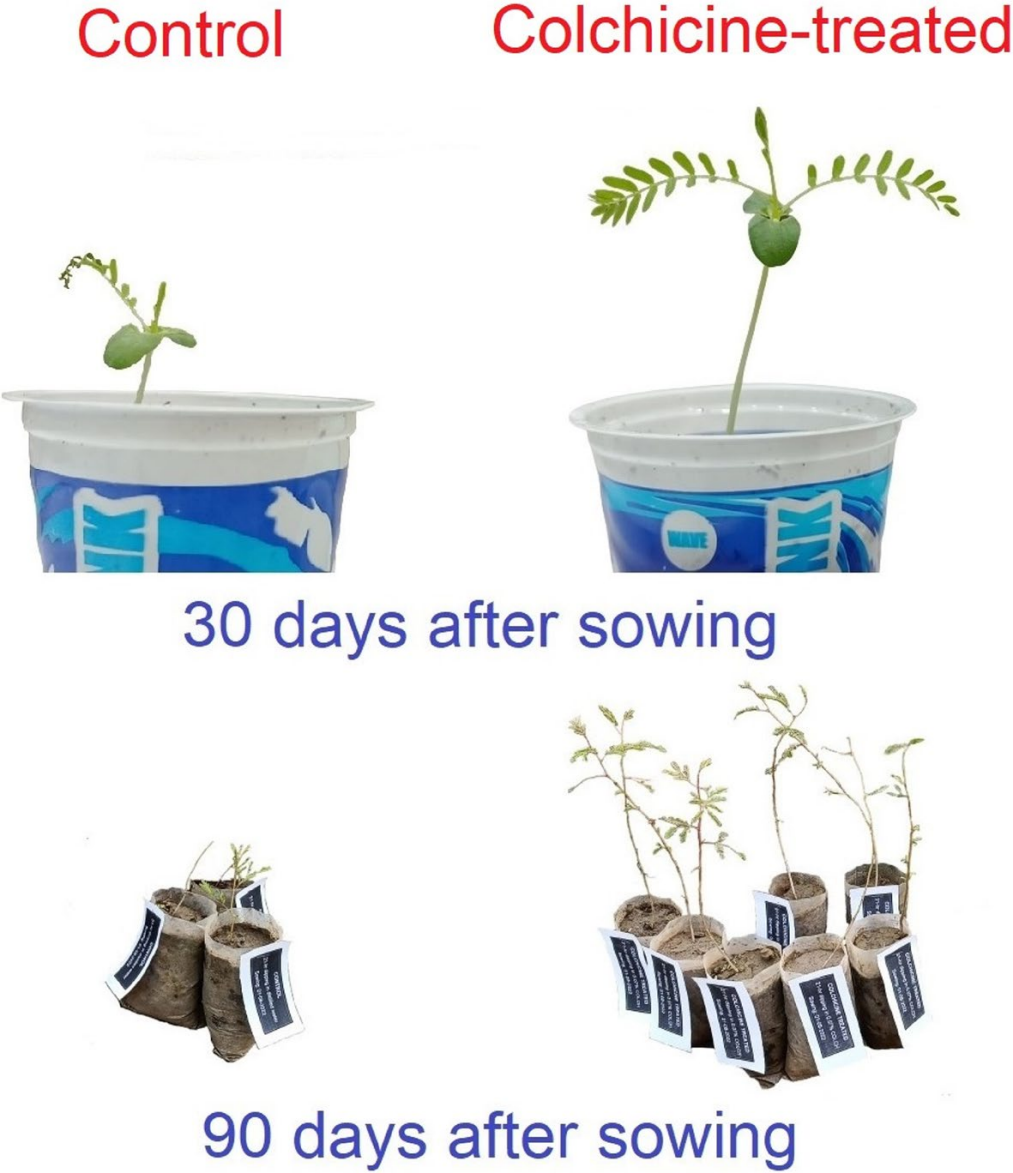
Visual difference between the colchicine seed treated (0.07% w/v for 21 h) and control seedlings of *Acacia nilotica* Linn. after 30 and 90 days of sowing

The experiment evidently illustrated significant differences (*P* ≤ *0.05*) for shoot length, root length, shoot fresh weight, root fresh weight, shoot dry weight, root dry weight, fresh biomass, stomatal size, stomatal density, and stomatal index under the main and interactive effects of seed treatment and time interval (Table 1). The shoot length of *A. nilotica* seedlings was about 1.8 times greater from colchicine-treated seeds than the control (Fig. 1A). In general, all the studied morphological and anatomical attributes showed a significant increase with the passage of the time interval, irrespective of treatments. For instance, the shoot length of *A. nilotica*, on the 6th week, was two-fold higher than on the 3rd week interval (Fig. 1A). The root length was approximately 53.5% higher in the seedlings raised from colchicine treated seeds compared to the control (Fig. 1B). The shoot and root fresh weights of seedlings under colchicine seed treatment were approximately 59.1% and 42.8% higher than control seedlings (Fig. 1C, D). Similarly, the shoot and root dry weights were around 51.5% and 40% higher in the seedlings grown under colchicine seed treatment compared to control ones (Fig. 1E, F). The fresh biomass of *Acacia nilotica* seedlings was also nearly 23.6% greater under colchicine seed treatment than control (Fig. 1G). Similarly, colchicine treated seeds produce seedlings with 35.9% higher stomatal size (Fig. 1H), 41.7% higher stomatal density (Fig. 1I), and 51.2% increased stomatal index compared to the control seeds (Fig. 1J). Overall, a noticeable improvement was observed in the visual appearance of seedlings under colchicine (0.07%) seed treatment with the passage of time compared to the control group (Fig. 2).

**Table 1:** Analysis of variance for factors (Treatment, interval and their interaction) for shoot length (SL), root length (RL), shoot fresh weight (SFW), root fresh weight (RFW), shoot dry weight (SDW), root dry weight (RDW), fresh biomass, stomatal size, stomatal density, and stomatal index in *Acacia nilotica* recorded at three- and six- week interval in control and colchicine seed-treated seedlings

Source of variance	SL	RL	SFW	RFW	SDW	RDW	Fresh biomass	Stomatal size	Stomatal density	Stomatal index
	**Percentage of total variance**									
Treatment (T)	33.70**	18.89**	24.93**	18.67**	23.44**	17.68**	32.05**	21.40**	20.49**	25.13**
Interval (I)	52.10**	67.70**	63.90**	71.27**	63.93**	71.29**	61.15**	68.34**	68.85**	53.18**
T × I	10.02**	12.15**	9.92**	9.28**	9.09**	7.67**	4.70*	6.26*	7.77*	16.82**
Error	4.08	1.25	1.68	0.76	3.52	3.34	2.08	3.98	2.88	4.85

^*^ Significant at *P* ≤ *0.05*

^**^ Significant at *P* ≤ *0.01*

### Physiological attributes of *Acacia nilotica* Linn

**Fig. 3:**
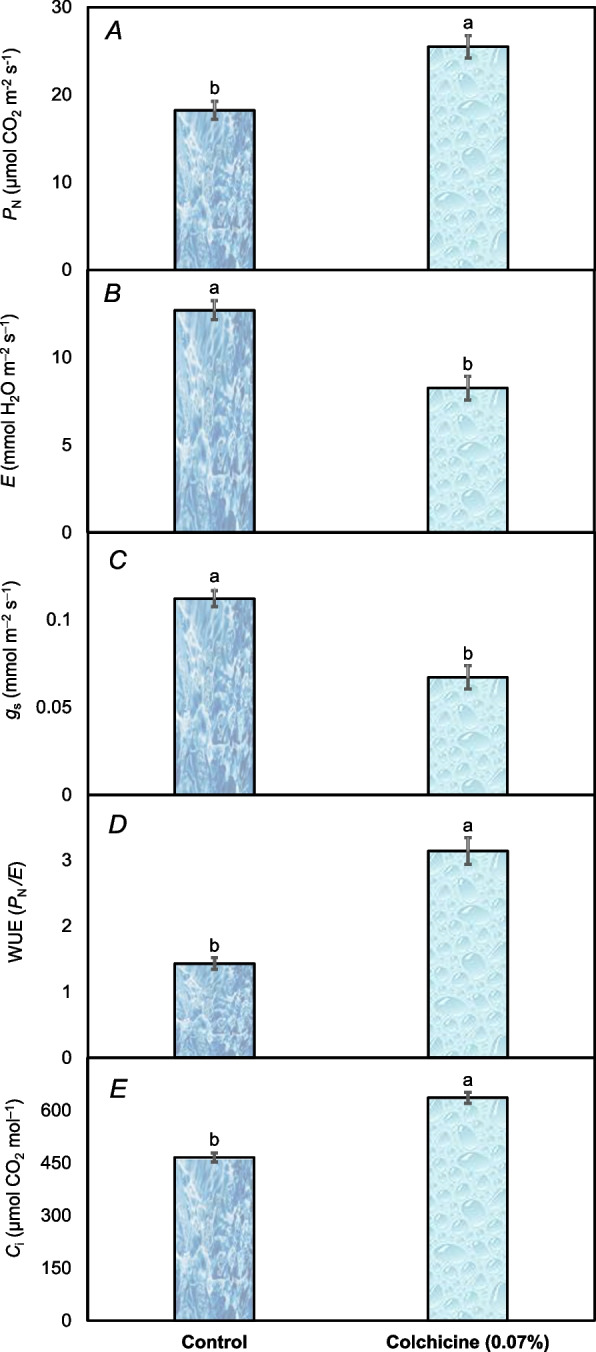
The physiological attributes including photosynthetic rate (*P*_N_) (**A**), transpiration rate (E) (**B**), stomatal conductance (g_s_) (**C**), water use efficiency (WUE) (**D**), and substomatal CO_2_ level (*C*_i_) (**E**) recorded in *Acacia nilotica*’ s seedlings raised under colchicine seed treatment and control. The bars indicate the standard error ( ±) of the mean (*n* = 3). Lettering denotes statistical variations between the treatment means carried out using Tukey’s HSD Test at the *P* ≤ *0.05* after analysis of variance

A significant effect on physiological attributes was recorded under colchicine seed treatment and control. Under colchicine seed treatment, the photosynthetic rate of *Acacia nilotica* seedlings was about 40% higher than those under control group (Fig. 3A). With colchicine seed treatment, the transpiration rate was also lower (8.25 mmol H_2_O m^–2^ s^–1^), almost a 54% decrease from control seedlings (12.7 mmol H_2_O m^–2^ s^–1^) was noted (Fig. 3B). Furthermore, the soaking of *Acacia nilotica*’ s seeds in colchicine resulted in a decreased stomatal conductance (0.067 mmol m^–2^ s^–1^) of seedlings comparably 67.1% than control (0.112 mmol m^–2^ s^–1^) (Fig. 3C). Following the colchicine seed treatment, the water use efficiency (WUE) of *Acacia nilotica*’ s seedlings increased from 1.43 to 3.14, reflecting a significant two-folds increase compared to control (Fig. 3D). Finally, the substomatal CO_2_ concentration was also found higher (465.2 μmol CO_2_ mol^–1^) in the seedlings grown under colchicine seed treatment rather than control (635.7 μmol CO_2_ mol^–1^) showcasing nearly a 36.7% comparable increase (Fig. 3E).

### Fluorescence-related attributes of *Acacia nilotica *Linn

**Fig. 4:**
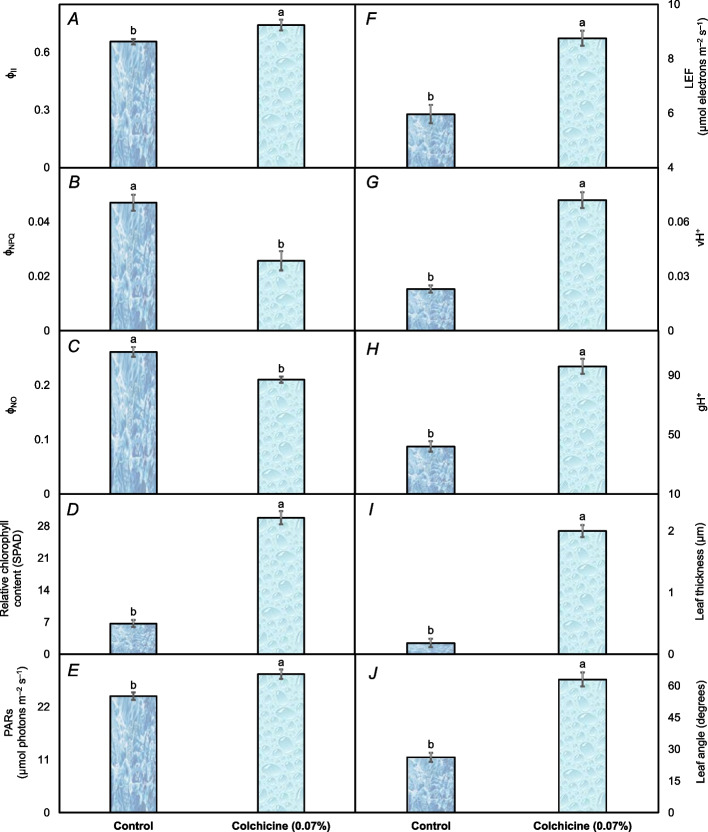
The chlorophyll fluorescence attributes including quantum yield of photosystem II (ф_II_) (**A**), non-photochemical quenching (ф_NPQ_) (**B**), non-regulatory energy dissipation (ф_NO_) (**C**), relative chlorophyll contents (**D**), photosynthetically active radiations (PARs) (**E**), linear electron flow (LEF) (**F**), proton flux (vH^+^) (**G**), proton conductivity (gH^+^) (**H**), leaf thickness (**I**), and leaf angle (**J**) recorded in *Acacia nilotica*’ s seedlings raised under colchicine seed treatment and control. The bars indicate the standard error ( ±) of the mean (*n* = 3). Lettering denotes statistical variations between the treatment means carried out using Tukey’s HSD Test at the *P* ≤ *0.05* after analysis of variance

The colchicine and control showed significant differences for all the studied fluorescence-related attributes. The quantum yield of photosystem II (Φ_II_) was 13.1% higher in the seedlings raised under colchicine seed treatment than those raised through the control seeds (Fig. 4A). Non-photochemical quenching (Φ_NPQ_) was noted around 83% lower in the seedlings grown under colchicine seed treatment than those grown under control conditions (Fig. 4B). The non-regulatory energy dissipation (Φ_NO_) was also found about 24% lower in the seedlings raised under colchicine seed treatment than the control’s seedlings (Fig. 4C). The seedlings raised under colchicine seed treatment had a 4.5 time rise in relative chlorophyll contents compared to control seedlings (Fig. 4D). The values of photosynthetically active radiations (PARs) were detected comparably 18.8% greater in the seedlings raised under colchicine seed treatment compared to the control (Fig. 4E). In terms of linear electron flow (LEF), the seedlings raised under colchicine seed treatment displayed about 47% increase in the electron’s movement within thylakoid membrane of chloroplasts than the control (Fig. 4F). The seedlings raised from colchicine treated seeds also induced three-fold stronger proton flux (vH^+^) than the control (Fig. 4G). In the case of proton conductivity (gH^+^), the seedlings under colchicine seed treatment exhibited approximately 2.3 times higher values than those under control conditions (Fig. 4H). Regarding leaf thickness, the seedlings raised under colchicine seed treatment also showed a three-fold increase compared to the control (Fig. 4I). After all, the leaf angle was also found greater (63°) in the seedlings raised under colchicine seed treatment than the control (26°) (Fig. 4J).

### Biochemical attributes of *Acacia nilotica* Linn

**Fig. 5:**
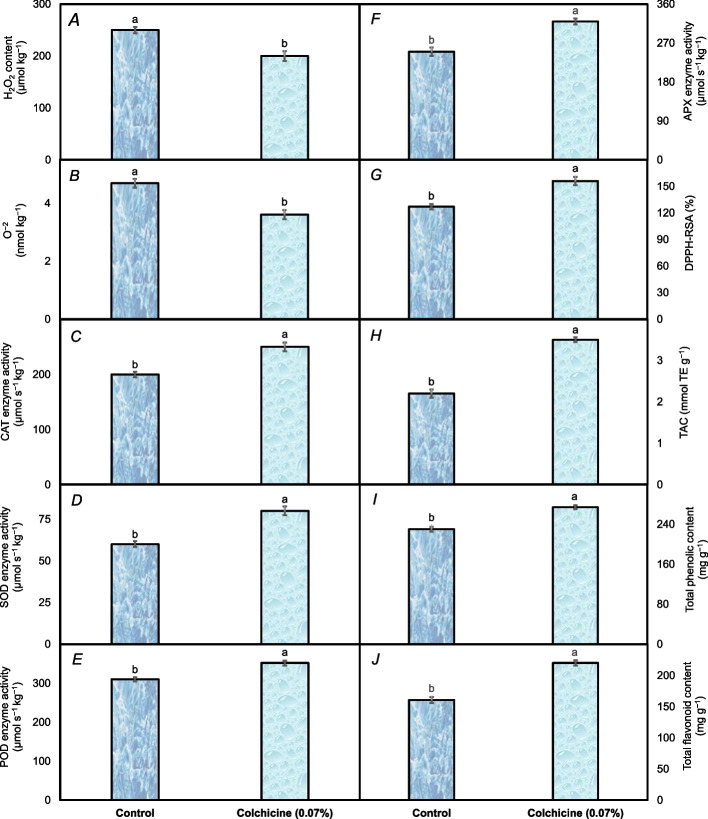
The biochemical attributes including hydrogen peroxide (H_2_O_2_) content (**A**) superoxide anion (O^−2^) (**B**), catalase (CAT) enzyme activity (**C**), superoxide dismutase (SOD) enzyme activity (**D**), peroxidase (POD) enzyme activity (**E**), Ascorbate peroxidase (APX) enzyme activity (**F**), 2,2-diphenyl-1-picrylhydrazyl-radical scavenging activity (DPPH-RSA) (**G**), total antioxidative capacity (TAC) (**H**), total phenolic content (**I**), and total flavonoid content (**J**) recorded in *Acacia nilotica*’ s seedlings raised under colchicine seed treatment and control. The bars indicate the standard error ( ±) of the mean (*n* = 3). Lettering denotes statistical variations between the treatment means carried out using Tukey’s HSD Test at the *P* ≤ *0.05* after analysis of variance

Colchicine seed treatment had a notable impact on the biochemical attributes of *A. nilotica* compared to the control group. Under colchicine seed treatment, the production of H_2_O_2_ content in the leaves of *A. nilotica* was approximately 25% lower than that under the control group (Fig. 5A). With colchicine seed treatment, the development of O^–2^ content was also lower (3.61 mmol kg^–1^); almost a 30% decline from control seedlings (4.69 mmol kg^–1^) was observed (Fig. 5B). On the other hand, the control group had lower catalase (CAT) enzyme activity (200 µmol s^–1^ kg^–1^) than the seedlings of *Acacia nilotica* grown from colchicine-soaked seeds (250 µmol s^–1^ kg^–1^) (Fig. 5C). Following the colchicine seed treatment, the superoxide dismutase (SOD) enzyme activity of *Acacia nilotica's* seedlings increased from 60 µmol s^–1^ kg^–1^ to 80 µmol s^–1^ kg^–1^, reflecting a significant 33% increase compared to control (Fig. 5D). The peroxidase (POD) enzyme activity was also found to be higher (352 µmol s^–1^ kg^–1^) in the seedlings grown under colchicine seed treatment rather than control (310 µmol s^–1^ kg^–1^), showcasing nearly a 13.5% comparable increase (Fig. 5E). The activity of ascorbate peroxidase (APX) was found to be comparably 28% greater in the seedlings raised under colchicine seed treatment compared to the control (Fig. 5F). The radical-scavenging activities of 2,2-diphenyl-1-picrylhydrazyl (DPPH) were found to be almost 23% higher in the seedlings raised from colchicine-treated seeds than the control ones (Fig. 5G). The seedlings raised after colchicine seed treatment also showed 59% greater total antioxidant capacity than control seedlings (Fig. 5H). The total phenolic content (TPC) of *Acacia nilotica* seedlings went up by almost 23% after being treated with colchicine (Fig. 5I). Finally, total flavonoid content was found to be approximately 37% higher in the seedlings from colchicine-treated seeds than the control (Fig. 5J).

## Discussion

**Fig. 6:**
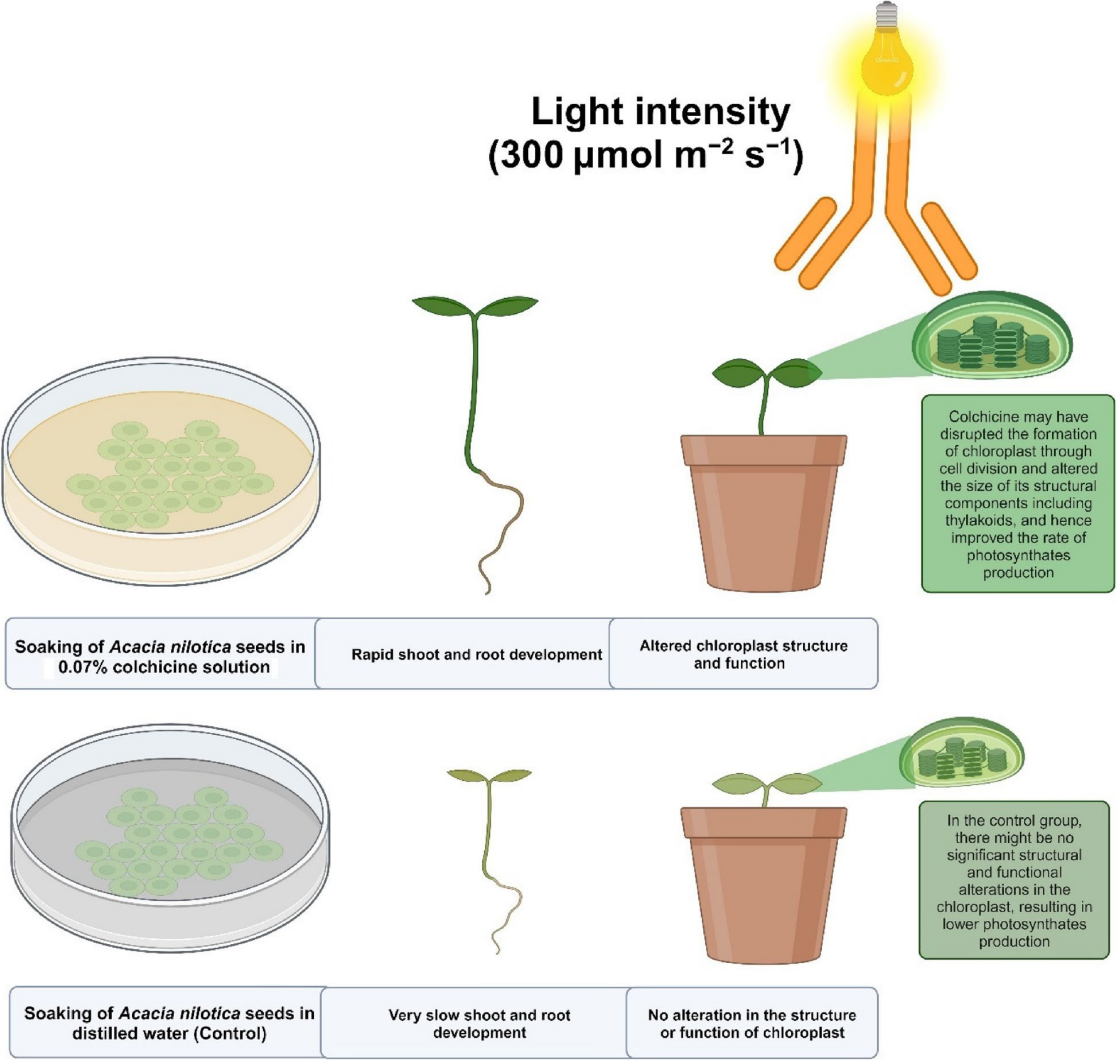
The proposed mechanism of phenotypic impact of colchicine seed treatment on *Acacia nilotica* Linn

**Fig. 7:**
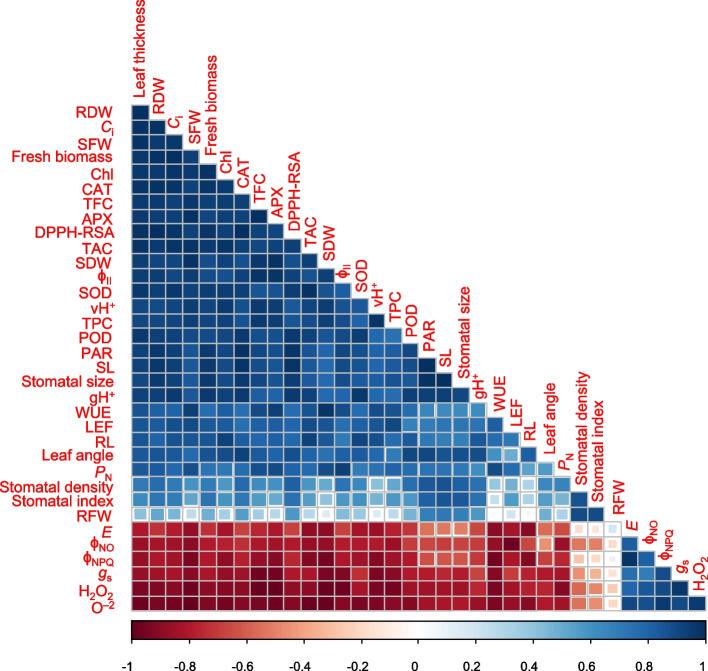
Pearson correlation (*P* ≤ *0.05*) among the studied attributes of *Acacia nilotica* Linn.; SL = shoot length, RL = root length, SFW = shoot fresh weight, RFW = root fresh weight, SDW = shoot dry weight, RDW = root dry weight, *P*_N_ = photosynthetic rate, *E* = transpiration rate, *g*_s_ = stomatal conductance, WUE = water use efficiency, *C*_i_ = substomatal CO_2_ level, ф_II_ = quantum yield of photosystem II, ф_NPQ_ = non-photochemical quenching, ф_NO_ = non-regulatory energy dissipation, Chl = relative chlorophyll content, PARs = photosynthetically active radiations, LEF = linear electron flow, vH^+^ = proton flux, gH^+^ = proton conductivity, CAT = catalase enzyme activity, SOD = superoxide dismutase enzyme activity, POD = peroxidase enzyme activity, APX = ascorbate peroxidase enzyme activity, DPPH-RSA = 2,2-diphenyl-1-picrylhydrazyl-radical scavenging activity, TAC = total antioxidant capacity, TPC = total phenolic content, TFC = total flavonoid content

This is the first ever report to the best of our knowledge, elucidating the mechanism of colchicine in expediting the growth and development of *Acacia nilotica* Linn. by improving its physiology and chlorophyll fluorescence (Fig. 6). According to Lam et al. (2014), healthy and vigorous plants can be produced from colchicine over 24 h. In this study, all the morphological and anatomical attributes were considerably enhanced by 0.07% colchicine seed treatment for a period of 21 h. The findings validate the results of Wang et al. [28] who observed a notable improvement in the germination percentage and shoot length of *Impatiens walleriana* after seed treatment with 0.05% colchicine for 48 h. Similarly, Li and Ruter [29] soaked seedlings of *Hibiscus moscheutos* at cotyledon stage and found a substantial increase in plant height and shoot fresh weight. In another study, Mori et al. [30] soaked seeds of *Limonium bellidifolium* in 0.05% colchicine solution for 72 h and found an enormous increase in germination percentage, shoot and root lengths as well as shoot and root fresh and dry weights. Likewise, He et al. [20] treated seeds of *Tagetes erecta* with a colchicine solution of 0.2% for 3 h and noted a boost in the morphological attributes compared to control. The positive strong relationship of shoot length, shoot fresh weight and fresh biomass with stomatal size, stomatal density, stomatal index, Φ_II_, vH^+^, gH^+^ and *P*_N_ suggests that the physiological robustness of seedlings contributed to their capacity for efficient photosynthesis by effectively using absorbed light energy and CO_2_ due to a greater rate of proton transport across cellular membranes and efficient ion transport processes which are crucial for various cellular functions and metabolic activities in vigorous seedlings (Fig. 7). The results are in agreement with those of Wei et al. [31] who found considerable differences for growth potential between untreated and colchicine (0.05–0.20%) applied seedlings of *Lespedeza formosa.* The significant influence of colchicine on morphological and anatomical attributes might be due to its direct effect on cell division which led to changes in cell size, hence resulted in the altered plant morphology and anatomy, including increased shoot and root length, shoot fresh weight, overall increased fresh biomass, increased stomatal size, stomatal density, and stomatal index.

The thick leaves containing a comparatively larger number of chloroplasts allow the plants to absorb much light as well as enhance subcellular CO_2_ diffusion within the leaves (Fig. 4I), which should encourage photosynthetic efficiency [32, 33]. In the current study, leaf thickness had a strong positive correlation with subcellular CO_2_ and the photosynthetic rate of *A. nilotica*'s seedlings (Fig. 7). The thickness of leaves is advantageous for photosynthesis, as chloroplasts, even those located deeper within the leaves, can absorb an ample supply of light [34]. Alternatively, leaf thickness had a strong negative correlation with *E* and *g*_s_ (Fig. 7) that are related to water transport [35]. The chlorophyll fluorescence is a measure of the light energy absorbed by chlorophyll molecules in plants, which is re-emitted as fluorescence light [36, 37]. It provides valuable insights into the photosynthetic performance of plants, including forest trees [38]. As chlorophyll fluorescence occurs in chloroplast, the process by which plants convert light energy into chemical energy [39]. So, chlorophyll fluorescence measurements are a valuable tool for assessing the effects of colchicine and understanding their physiological consequences on chloroplasts. In this study, leaf angle was recorded due to its significant role in the chlorophyll fluorescence of forest trees by influencing light absorption, distribution, and physiological responses of leaves to varying light conditions [40]. The leaves, which are oriented perpendicularly to incoming sunlight, receive the maximum amount of light, allowing chloroplasts to absorb more photons and initiate photosynthesis efficiently. In contrast, leaves at oblique angles receive less direct light, leading to reduced chlorophyll excitation and lower rates of photosynthesis [41]. Photosynthetically active radiation (PARs) is also an important photosynthetic attribute, indicating a fraction of the incoming light (400–700 nm) that can be utilized for photosynthesis. In the present study, leaf angle showed a strong positive correlation with PARs, indicating that the leaves angled wider towards the incident light received more PARs, hence enhancing the photosynthesis process. The findings are in agreement with those of Yirdaw and Luukkanen [42], who evaluated the transmittance rate of PARs in five different forest tree species and found a decisive role of leaf angle in the light absorption for photosynthesis. The positive relationship of leaf thickness with Φ_II_ shows that seedlings with thick leaves may have many chloroplasts per unit leaf area (Fig. 7). An increase in Φ_II_ with an increase in leaf thickness has been reported in many previous studies [43–45]. A tremendous increase in the leaf thickness was also noticed in the plants of *Gladiolus grandiflorus* subjected to a 0.3% colchicine treatment compared to the control [22].

According to Hosseini and Javanbakht [46], the induction of tetraploidy in the plants involves notable modifications in biochemical attributes, including a substantial decrease in reactive oxygen species (ROS) and an increase in TAC, TPC, and the activities of antioxidative enzymes including CAT, SOD, POD, and APX. In the current study, a significant decrease in H_2_O_2_ and O^–2^ while an increase in CAT, SOD, POD, APX, DPPH-RSA, TAC, TPC, and TFC was observed in the leaves of *A. nilotica*. The results of our study support the hypothesis of Hosseini and Javanbakht [46]. Our findings are also in good agreement with those of Akbari et al. [47], who found a substantial improvement in the activities of APX and CAT enzymes as well as phenolics and carotenoids in the leaves of *Carum copticum* after seed treatment with 0.5 g L^–1^ colchicine for 6 h. Our findings are also validated by previous findings of Keshmiri et al. [48], who found a significant increase in the production of TFC in *Linum album*’s callus during chromosome doubling. Thus, our research may be useful in elaborating the mechanism of colchicine’s morpho-physiological impact on *A. nilotica.*

In the present study, our measurements were limited to phenotypic and physiological attributes. Further molecular studies are required to explore the changes occurred in the ploidy level underlying the observed improvements in growth and development to gain insights into molecular mechanism and potential genetic modifications for enhancing *A. nilotica*.

## Conclusion

The optimized concentration of colchicine treatment (0.07%) significantly enhanced *Acacia nilotica*'s growth, physiology, and biochemical properties, presenting a promising approach for efficient seed-based propagation. Future research should focus on scaling this method for field applications, considering environmental implications and long-term effects to establish sustainable cultivation practices for *A. nilotica*.

## Methodology

### Seed source

The seeds of *Acacia nilotica* Linn., were collected from the Forestry Research Area (29°37′17.4′′ N 71°76′53.6′′ E), The Islamia University of Bahawalpur (IUB), Pakistan and transported to Plant Tissue Culture Laboratory, Department of Horticultural Sciences (DoHS), IUB to carry out the research trial.

### Colchicine seed treatment

In the present experiment, a total of 30 seeds were dipped in each of the three replicates of colchicine treatment (0.07%) and distilled water (control) on August 1, 2022, for 21 h. The selection of a 0.07% colchicine concentration was based on a preliminary assessment involving solutions with concentrations of 0%, 0.035%, 0.07%, 0.14%, and 0.28%. The immersion duration was also chosen based on preliminary investigations, during which 7 h, 14 h, 21 h, and 28 h of durations were tested.

### Experimental protocol

The seeds for each colchicine and control group were sterilized in 70% ethanol solution for 30 s and washed five times in double distilled water. Then seeds were soaked in the optimized solution colchicine solution (0.07%). After drying them out for 24 h, each seed was sown in an individual disposable cup containing silt as a growing media, and the seeds were allowed to germinate and develop in growth room of Plant Tissue Culture Laboratory, DoHS, IUB. The temperature and humidity conditions in the growth room were maintained to 21 °C and 62%, respectively. The photoperiod was maintained through the provision of 16 h of daylight and an 8 h of darkness with the photosynthetic photon flux density of 300 μmol m^−2^ s^−1^ light. The seedlings were shifted in plastic bags 35 days after sowing.

### Measurement of attributes

The data related to morphometric, anatomical, physiological, fluorescence, and biochemical analysis of *Acacia nilotica* Linn. in response to seed treatment with colchicine solution and distilled water (control) were recorded on 21st and 42nd day of sowing for 20 randomly selected seedlings from each experimental unit.

### Morphometric and anatomical attributes

The number of germinated seeds were recorded on a daily basis and their percentage was calculated and number of days to 80% germination were also computed. The values of shoot and root length were taken at 3rd and 6th week of sowing from every 20 seedlings and their average was calculated. Vigor index was calculated using the below formula as adopted by Zhu and Hong [49].

$$Seedling\ vigor\ index \left(SVI\right)={\text{S}}\times \sum \frac{Gt}{Dt}$$

where S is seedling height of the 14th day, Gt is number of germinated seeds in the “tth” day, Dt is number of days from the first day to the “tth” day. The fresh weight of biomass including shoots and roots as well as their dry weights were taken using an analytical balance (PR Series, Ohaus, Newark, USA). For heat drying of both shoots and roots, they were initially air-dried in the sun until most of the moisture had escaped. Subsequently, they were placed in a hot dry oven (SLN 15 SMART, Pol-Eko, Wodzisław Śląski, Poland) at 65°C until their dry mass became constant.

Three- and six-week-old seedlings of treated and untread *A. nilotica* were taken for counting stomata under an optical microscope (B-192, Optika, Ponteranica, Italy) and a 0.5 mm^2^ acetate template was used. Stomatal density *i.e.*, number of stomata mm^−2^ and stomatal index were also recorded at three- and six-week interval. The stomatal index was determined by using the below formula [50]:

$$Stomatal\ index=\frac{Stomatal\ density}{Stomatal\ density+Epidermal\ cells\ density}\times 100$$

### Physiological attributes

The physiological parameters, including photosynthetic rate (μmol CO_2_ m^–2^ s^–1^), transpiration rate (mmol H_2_O m^–2^ s^–1^), stomatal conductance (mol H_2_O m^–2^ s^–1^), and intercellular CO_2_ concentration (μmol CO_2_ mol^–1^), were measured on the 21st and 42nd days after sowing using an infrared gas analyzer (IRGA) (model LCi-SD, manufactured by ADC Bio-scientific in England). Then, the mean values were calculated from the readings of both intervals. The measurement was conducted by selecting three fully developed and healthy leaves of 20 randomly selected plants from each experimental unit. The IRGA measurements were taken under specific conditions including a light intensity of 300 µmol m^–2^ s^–1^, a leaf surface temperature ranging from 31.7°C to 36.5°C, a leaf surface area of 6.25 cm^2^, a CO_2_ concentration of 390.12 µmol^–1^, an airflow rate per unit area of leaf (U) at 200.9 µmol s^–1^, an atmospheric pressure (P) of 991 mBar, and a H_2_O partial pressure of 13.4 mBar. Water use efficiency (WUE) was calculated using the below formula:

$$Water\ use\ efficiency (WUE)=\frac{Photosynthetic\ rate\ ({P}_{{\text{N}}})}{Transpiration\ rate\ (E)}$$

### Fluorescence-related attributes

The fluorescence measurements in *Acacia nilotica* plants were taken on the 21st and 42nd day of sowing, using a MultispeQ-Beta instrument and the PhotosynQ platform software [51] and average was taken from the readings of both intervals. These attributes encompassed quantum yield of photosystem II (Φ_II_), relative chlorophyll contents, photosynthetically active radiations (PARs), non-photochemical quenching (Φ_NPQ_), non-regulatory energy dissipation (Φ_NO_), proton flux (vH^+^), proton conductivity (gH^+^), linear electron flow (LEF), leaf thickness, and leaf angle.

### Biochemical attributes

The biochemical attributes in *Acacia nilotica* seedlings were taken on the 21st and 42nd day of sowing, and average was computed from the readings taken at both intervals. To determine the content of H_2_O_2_, the method described by Haider et al. [52] was followed. In this approach, 1 g of *A. nilotica'*s leaf disc was homogenized in 1 ml of 0.1% TCA and then centrifuged for 15 min at a speed of *12,000* × *g*. Subsequently, 0.5 ml of the extracted supernatant was combined with 10 mmol L^−1^ of phosphate buffer (pH = 7) and 1M KI. Afterwards, the measurement was obtained by calculating the absorbance of each sample at a wavelength of 390 nm and expressed as μmol kg^−1^ FW.

The quantity of O^−2^ in leaf tissues of *A. nilotica* was examined utilizing a technique documented by Hasan et al. [53]. One gram of *A. nilotica*'s leaf disc was thoroughly blended with 3 ml of phosphate buffer containing 1% polyvinylpyrrolidone at 4°C. Subsequently, the samples underwent centrifugation with a force of (*10,000* × *g*) for a duration of 15 min. They were combined thereafter with a solution containing 10 mmol L^–1^ hydroxylamine hydrochloride and allowed to react at a temperature of 25°C for a duration of 30 min. The absorption of each sample was observed at a wavelength of 530 nm. The quantity of O^−2^ was calculated by utilizing the NO_2_ curve as a reference, and the measurements were expressed in nmol kg^−1^ FW.

For the determination of antioxidative enzymes, a leaf sample of *A. nilotica* weighing 1 g was ground using a cold mortar and pestle in 2 ml of phosphate buffer with a pH of 7.2. The mixture was subsequently subjected to centrifugation using a Rotofix 46 centrifuge (Hettich, Kirchlengern, Germany) at a speed of *10,000* × *g* for a duration of five minutes at a temperature of 4°C. The activities of antioxidative enzymes were measured after collecting the supernatant. The activities of catalase (CAT) (EC 1.11.1.6), superoxide dismutase (SOD) (EC 1.15.1.1), peroxidase (POD) (EC 1.11.1.7), and ascorbate peroxidase (APX) (EC 1.11.1.11) were measured using the method outlined previously by Haider et al. [52]. The samples were analyzed at different wavelengths: 240 nm for CAT, 560 nm for SOD, 470 nm for POD, and 290 nm for APX. The enzyme activities were quantified in µmol kg^−1^ FW.

The 2,2-diphenyl-1-picrylhydrazyl (DPPH) radical scavenging activity was determined using the method as previously described by Ali et al. [4], and results were represented as a percentage of inhibition. For the determination of the total antioxidant capacity of *A. nilotica*'s seedlings, the methodology reported by Osman et al. [54] was followed. The molybdate reagent consisted of 1 ml of 0.6 M H_2_SO_4_, 28 mM Na_3_PO_4_, and 4 mM (NH_4_)_2_MoO_4_. The volume was then increased to 50 ml by adding distilled H_2_O. The leaves of *A. nilotica* were extracted following the homogenization process. The resulting supernatant layer of the extract, measuring 100 ml, was then transferred into a test tube. This test tube already included 3 ml of distilled water and 1 ml of molybdate reagent. The test tube was subjected to incubation at a temperature of 95°C for a duration of 90 min. Subsequently, the test tube was allowed to cool until it reached the ambient temperature, which took around 20–30 min. The absorbance of the resulting reaction mixture was then measured at a wavelength of 695 nm. The mean values were recorded, and the results were reported in micromoles equivalents of Trolox per gram of fresh leaf weight of the sample.

The estimation of the total phenolic content (TPC) in the leaves of *A. nilotica* was carried out by measuring the absorbance at a wavelength of 765 nm using the Folin-Ciocalteu reagent [55]. A standard curve was established for gallic acid, and the content of total phenolics (TPC) was expressed in mg kg^−1^. The measurement of flavonoid content was accomplished using the methodology adopted by Kaushik et al. [56]. To summarise, a 1 ml sample of *A.* *nilotica*'s leaf extract was mixed well with 4 ml of deionized water and 300 μl of NaNO_2_. The samples were subsequently stored for a duration of 5 min. After that, 300 µl of AlCl_3_ was introduced into a solution containing 2 ml of 1M NaOH. The absorbance was then measured at a wavelength of 510 nm. It was denoted as mg kg^−1^.

### Statistics

Microsoft Excel 2016 was used for data processing. Data analysis involved the application of analysis of variance (ANOVA) using Statistix 9® for Windows (Analytical Software, Tallahassee, USA), with subsequent comparisons of mean values carried out using the least significant difference and Tukey’s HSD Tests. The correlation among the measured attributes was examined using “corrplot” function of R program 4.0.2 through the general linear model procedure [57]. A significance level of 5% was chosen for all the above statistics.

### Supplementary Information

Supplementary Material 1.

## Data Availability

All the data related to this work can be sourced from the corresponding authors.

## Abbreviations

ANOVA: Analysis of variance
APX: Ascorbate peroxidase
CAT: Catalase
*C*_i_: Sub-stomatal CO_2_ level
DoHS: Department of Horticultural Sciences
DPPH-RSA: 2,2-Diphenyl-1-picrylhydrazyl-radical scavenging activity
DTG_80_: Days to 80% germination
*E*: Transpiration rate
gH^+^: Proton conductivity
*g*_s_: Stomatal conductance
IUB: The Islamia University of Bahawalpur
LEF: Linear electron flow
PARs: Photosynthetically active radiations
*P*_N_: Photosynthetic rate
POD: Peroxidase
SOD: Superoxide dismutase
SPAD: Soil and plant analysis development
SVI: Seedling vigor index
TAC: Total antioxidant capacity
TFC: Total flavonoid content
TPC: Total phenolic content
Tukey’s HSD test: Tukey's honestly significant difference test
vH^+^: Proton flux
WUE: Water use efficiency
Φ_II_: Quantum yield of photosystem II
Φ_NO_: Non-regulatory energy dissipation
Φ_NPQ_: Non-photochemical quenching
