# Supplementary material for: Exploring the mechanism of transformation in Acacia nilotica (Linn.) triggered by colchicine seed treatment
Source: BMC Plant Biol. 2024 May 21;24:428. doi: 10.1186/s12870-024-05139-9 (PMC11106899; doi:10.1186/s12870-024-05139-9)
Supplement: Supplementary file 1 — Supplementary Material 1. [file 12870_2024_5139_MOESM1_ESM.docx]

1. **Selection criteria for colchicine concentration during preliminary assessment**

The optimization of colchicine treatment levels was carried out based on selected morphological attributes such as germination percentage, seedling vigor index, shoot length, root length, and fresh biomass.

- 1. **Germination percentage and Seedling vigor index**

**Fig. S1.** Optimization of colchicine treatment level for germination percentage and seedling vigor index of *Acacia nilotica*. The bars indicate the standard error (±) of the mean (n = 3). Lettering denotes statistical variations between the treatment means carried out using Least Significant Difference Test at the *P ≤ 0.05* after analysis of variance.

- 1. **Shoot and root lengths**

**Fig. S2.** Optimization of colchicine treatment level for shoot length and root length of *Acacia nilotica*. The bars indicate the standard error (±) of the mean (n = 3). Lettering denotes statistical variations between the treatment means carried out using Least Significant Difference Test at the *P ≤ 0.05* after analysis of variance.

- 1. **Fresh biomass**

**Fig. S3.** Optimization of colchicine treatment level for fresh biomass of *Acacia nilotica*. The bars indicate the standard error (±) of the mean (n = 3). Lettering denotes statistical variations between the treatment means carried out using Least Significant Difference Test at the *P ≤ 0.05* after analysis of variance.

1. **Seed Viability Test**

**Fig. S4.** Percentage of germinated and non-germinated seeds of *Acacia nilotica*. The bars indicate the standard error (±) of the mean (n = 3).

1. **Physicochemical analyses of soil and water**

| **Particular** | **Unit** | **Values** |
| --- | --- | --- |
| Soil texture | - | Sandy loam |
| Sand | % | 61 |
| Silt | % | 23 |
| Clay | % | 16 |
| Saturation | % | 27 |
| pH | - | 8.1 |
| EC | dS m^–1^ | 1.9 |
| Total dissolved salts | ppt | 0.87 |
| Organic matter | % | 0.56 |
| CEC | c mol kg^–1^ | 8.6 |
| Total N | % | 0.048 |
| Available P | mg L^–1^ | 0.62 |
| Available K | mg L^–1^ | 83 |

**Table S1.** Physicochemical analysis of the soil used in the experiment.

| **Particular** | **Unit** | **Value** |
| --- | --- | --- |
| pH | - | 7.2 |
| EC | dS m^–1^ | 1.03 |
| Total dissolved salts | g L^–1^ | 0.98 |
| Ca + Mg | Meq L^–1^ | 7.14 |
| Na | Meq L^–1^ | 4.75 |
| CO_3_ | Meq L^–1^ | - |
| HCO_3_ | Meq L^–1^ | 2.44 |
| Cl | Meq L^–1^ | 0.85 |
| Sodium adsorption ratio | - | 2.40 |
| Residual sodium carbonate | Meq L^–1^ | 0.28 |

**Table S2.** Physicochemical analysis of irrigated water used in the experiment.
